# Integrative Analysis of a Cross-Loci Regulation Network Identifies *App* as a Gene Regulating Insulin Secretion from Pancreatic Islets

**DOI:** 10.1371/journal.pgen.1003107

**Published:** 2012-12-06

**Authors:** Zhidong Tu, Mark P. Keller, Chunsheng Zhang, Mary E. Rabaglia, Danielle M. Greenawalt, Xia Yang, I-Ming Wang, Hongyue Dai, Matthew D. Bruss, Pek Y. Lum, Yun-Ping Zhou, Daniel M. Kemp, Christina Kendziorski, Brian S. Yandell, Alan D. Attie, Eric E. Schadt, Jun Zhu

**Affiliations:** 1 Institute of Genomics and Multiscale Biology, Mount Sinai School of Medicine, New York, New York, United States of America; 2 Department of Genetics and Genomic Sciences, Mount Sinai School of Medicine, New York, New York, United States of America; 3 Department of Biochemistry, University of Wisconsin–Madison, Madison, Wisconsin, United States of America; 4 Merck Research Laboratories, Boston, Massachusetts, United States of America; 5 Department of Integrative Biology and Physiology, University of California Los Angeles, Los Angeles, California, United States of America; 6 Merck Research Laboratories, Rahway, New Jersey, United States of America; 7 Department of Genetics, Rosetta Inpharmatics, Merck, Seattle, Washington, United States of America; 8 Department of Biostatistics and Medical Informatics, University of Wisconsin–Madison, Madison, Wisconsin, United States of America; 9 Department of Statistics, University of Wisconsin–Madison, Madison, Wisconsin, United States of America; 10 Graduate School of Biological Sciences, Mount Sinai School of Medicine, New York, New York, United States of America; 11 Pacific Biosciences, Menlo Park, California, United States of America; University of Oxford, United Kingdom

## Abstract

Complex diseases result from molecular changes induced by multiple genetic factors and the environment. To derive a systems view of how genetic loci interact in the context of tissue-specific molecular networks, we constructed an F2 intercross comprised of >500 mice from diabetes-resistant (B6) and diabetes-susceptible (BTBR) mouse strains made genetically obese by the *Leptin^ob/ob^* mutation (*Lep^ob^*). High-density genotypes, diabetes-related clinical traits, and whole-transcriptome expression profiling in five tissues (white adipose, liver, pancreatic islets, hypothalamus, and gastrocnemius muscle) were determined for all mice. We performed an integrative analysis to investigate the inter-relationship among genetic factors, expression traits, and plasma insulin, a hallmark diabetes trait. Among five tissues under study, there are extensive protein–protein interactions between genes responding to different loci in adipose and pancreatic islets that potentially jointly participated in the regulation of plasma insulin. We developed a novel ranking scheme based on cross-loci protein-protein network topology and gene expression to assess each gene's potential to regulate plasma insulin. Unique candidate genes were identified in adipose tissue and islets. In islets, the Alzheimer's gene *App* was identified as a top candidate regulator. Islets from 17-week-old, but not 10-week-old, *App* knockout mice showed increased insulin secretion in response to glucose or a membrane-permeant cAMP analog, in agreement with the predictions of the network model. Our result provides a novel hypothesis on the mechanism for the connection between two aging-related diseases: Alzheimer's disease and type 2 diabetes.

## Author Summary

Alzheimer's disease and type 2 diabetes are two common aging-related diseases. Numerous studies have shown that the two diseases are associated. However, the mechanisms of such connection are not clear. Both diseases are complex diseases that are induced by multiple genetic factors and the environment. To understand the molecular network regulated by complex genetic factors causing type 2 diabetes, we constructed an F2 intercross comprised of >500 mice from diabetes-resistant and diabetic mouse strains. We measured genotypes, clinical traits, and expression profiling in five tissues for each mouse. We then performed an integrative analysis to investigate the inter-relationship among genetic factors, expression traits, and plasma insulin, a hallmark diabetes trait, and developed a novel method for inferring key regulators for regulating plasma insulin. In islets, the Alzheimer's gene *App* was identified as a top candidate regulator. Islets from 17-week-old, but not 10-week-old, *App* knockout mice showed increased insulin secretion in response to glucose, in agreement with the predictions of the network model. Our result provides a novel hypothesis on the mechanism for the connection between two aging-related diseases: Alzheimer's disease and type 2 diabetes.

## Introduction

Complex diseases, such as diabetes and obesity, result from the interaction of genetic and environmental factors [1]–[3]. Approximately 170 gene loci have been robustly implicated in diabetes through genome-wide association studies [4]. Studies with knockout mouse models have identified hundreds of genes that can act autonomously to regulate insulin levels (MP:0001560) [5]. However, it is still elusive to understand the underlying mechanisms of how these loci or genes contribute to diseases.

Network modeling methods have been developed based on the premise that complex diseases are often caused by perturbation to a sub-network of genes [1], [6]–[14]. We have applied these methods to identify causal genes for diabetes-related traits in multiple experimental mouse crosses [13]–[14] and human populations [1]. These analyses suggest that potentially many thousands of genes, under the right circumstances, can affect metabolic states.

With the advancement of high-throughput technologies, such as DNA and RNA sequencing, methods that integrate various high-volume data sources are providing for more comprehensive characterizations of biological systems [15]–[18]. New methods have been developed to utilize high-dimensional data sets to infer unknown pathways, untangle gene-based regulatory networks, and identify novel disease-causing genes [13], [19]–[23]. However, studying complex diseases at a systems level is still in its infancy. New technologies for data collection and novel methodologies of data interpretation are needed for a better resolution view of the system.

In this study, we developed a network-based model to identify key genes that regulate plasma insulin levels in a B6XBTBR obese F2 cross. By applying a causality test for genes whose expression trait is linked to two loci that overlap insulin QTLs (quantitative trait loci) and integrating protein-protein interactions, we constructed a network for each of five tissues under study. We predicted that multiple genes in the pancreatic islet network may be involved in modulating plasma insulin levels in the B6XBTBR F2 cross, including *App*, *Gria3*, *Grb10*, *Calca*, and *Ins1*. In particular, our pancreatic islet network predicts that the Alzheimer's disease gene, amyloid precursor protein *App* is a negative regulator of insulin abundance in the plasma. We therefore studied insulin secretion from islets of *App* knockout mice. Islets from 17-wk-old, but not 10-wk-old *App −/−* mice showed an increase in glucose and cAMP-stimulated insulin secretion, confirming that *App* acts as a negative regulator of insulin secretion. This result elucidates a possible mechanism connecting two common age-related diseases, Alzheimer's disease and type 2 diabetes.

## Results

We generated an F2 inter-cross between diabetes-resistant (B6) and diabetes-susceptible (BTBR) mouse strains, made genetically obese in response to the *Lep^ob^* mutation [24]. The cross consisted of >500 mice, evenly split between males and females. A comprehensive set of ∼5000 genotype markers were used to genotype each F2 mouse (∼2000 informative SNPs were used for analysis), and the expression levels of ∼40 K transcripts (corresponding to 25,901 unique genes) were monitored in five tissues (adipose, liver, pancreatic islets, hypothalamus, and gastroc (gastrocnemius muscle)) that were harvested from each mouse at 10 weeks of age. In addition to gene expression, several key T2D-related traits were determined for each mouse. The medians, and 1st and 3rd quartiles for the following traits: body weight, the number of islets harvested per pancreas, HOMA, plasma insulin, glucose, triglyceride, and C-peptide are listed in Table 1.

**Table 1:** Diabetes-related clinical traits for 275 B6XBTBR-ob/ob F2 male mice at 10 weeks of age.

Trait	Median	1st quartile	3rd quartile
body weight (gm)	52	47	55
plasma glucose (mg/dl)	529	460	598
plasma insulin (ng/ml)	6.4	3.8	12.5
HOMA (insulin×glucose/22.5)	156	92	281
plasma C-peptide (nM)	3.5	2.5	4.8
plasma TG (mg/dl)	228	164	327
# islets per pancreas	184	118	268

All metrics were made following a 4 hour fasting period. The number of islets per pancreas is that collected for each mouse following pancreatic collagenase digestion and manual hand-picking as described [24].

**Figure 1:**
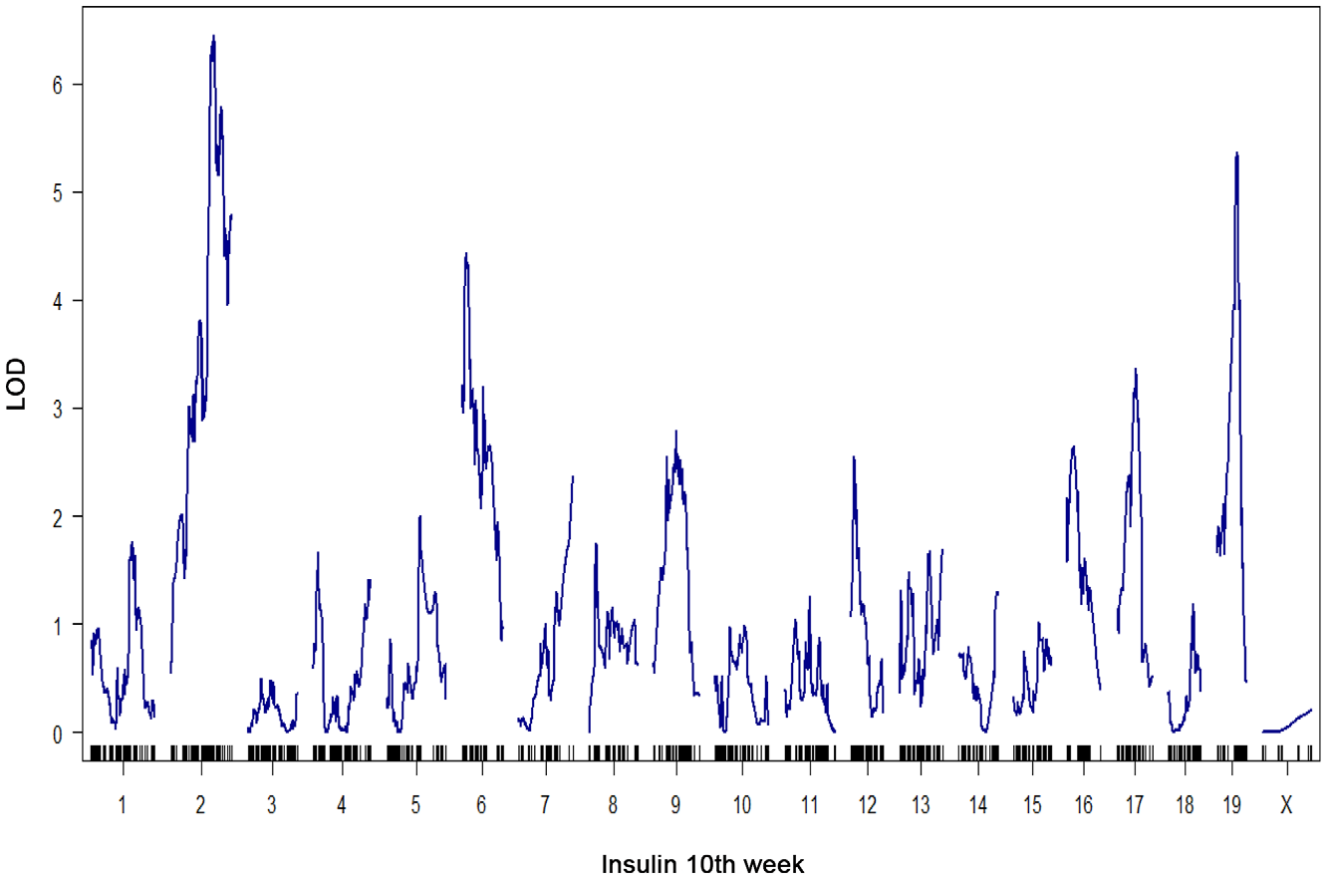
Genome-wide linkage profile for plasma insulin at 10-week-old BTBRx B6 *ob/ob* F2 male mice. The top two QTLs are chromosome 2 (LOD = 6.5) and chromosome 19 (LOD = 5.4). We select these two loci to study the underlying protein-protein interaction networks in various tissues that could explain their joint regulation on insulin trait.

At a genome-wide p-value<0.05, plasma insulin shows significant linkage to multiple loci in male mice, including chromosomes 2 at 59.5–82.5 cM, 6 at 0–33.66 cM, and 19 at 25–35.38 cM with LOD scores of ∼6.5, 4.4, and 5.4, respectively (Figure 1). Using linear regression modeling, the loci on chromosomes 2, 6, and 19 explain 10.6%, 6.0%, and 8.4% of the variation of plasma insulin, respectively. The top two loci at chromosomes 2 and 19 jointly explain 16.8% of the variance (Figure S1). The top two loci are significant at genome-wide p-value<0.01, and are consistent with the results of another independent F2 cross from the same two strains Stoehr *et al.* [25].

To elucidate the gene-gene interactions underlying the heritability of plasma insulin, we examined gene expression profiles in several key tissues and extended the causality method to construct a protein interaction network for genes with expression quantitative trait loci, or eQTLs linked to insulin loci. To simplify the interaction models, we modeled the effects of two loci. Further analysis focused on interactions of genes with eQTLs linked to the top two plasma insulin loci, at chromosomes 2 and 19. We hypothesize that the joint regulation of plasma insulin at the two QTLs is mediated by gene-gene interactions whose expression variations are linked to the two loci. We further developed a novel ranking algorithm to infer candidate genes for regulation of plasma insulin.

### Identifying genes with overlapping eQTLs with the insulin QTLs

Treating gene expression as a phenotypic trait, we computed eQTLs, for all genes expressed in pancreatic islets, white adipose tissue, liver, hypothalamus, and gastroc muscle of each male F2 mouse. We hypothesized that genes with eQTLs that co-localized with insulin QTLs are co-regulated by common genetic factors [13]. We identified eQTLs within each tissue that had LOD profile peaks on chromosomes 2 and 19, the same genomic regions containing the peak insulin linkages. Among genes physically located within the insulin QTLs on chromosome 2 or 19 (Table S1), 89 genes have cis-eQTLs (gene expression QTLs are mapped to within 10 Mb of the genomic location of the genes) in islet, 66 in white adipose tissue, 52 in liver, 51 in hypothalamus, and 5 in gastroc have cis-eQTLs. Clearly, genes with cis-eQTLs may play significant roles in modulating insulin and methods have been developed to identify the causal genes with cis-eQTLs for various phenotypic traits [14], [26]–[27]. However, each gene with a cis-eQTL can only explain the variance in the trait linked to its location. Here we considered a complementary strategy where we focused on genes with *trans*-eQTLs and interactions among them that integrate perturbations from multiple loci. As a greater number of genes showed *trans*-linkage, it is worth studying the potential mechanisms by which these genes jointly mediate the phenotypic variation.

**Figure 2:**
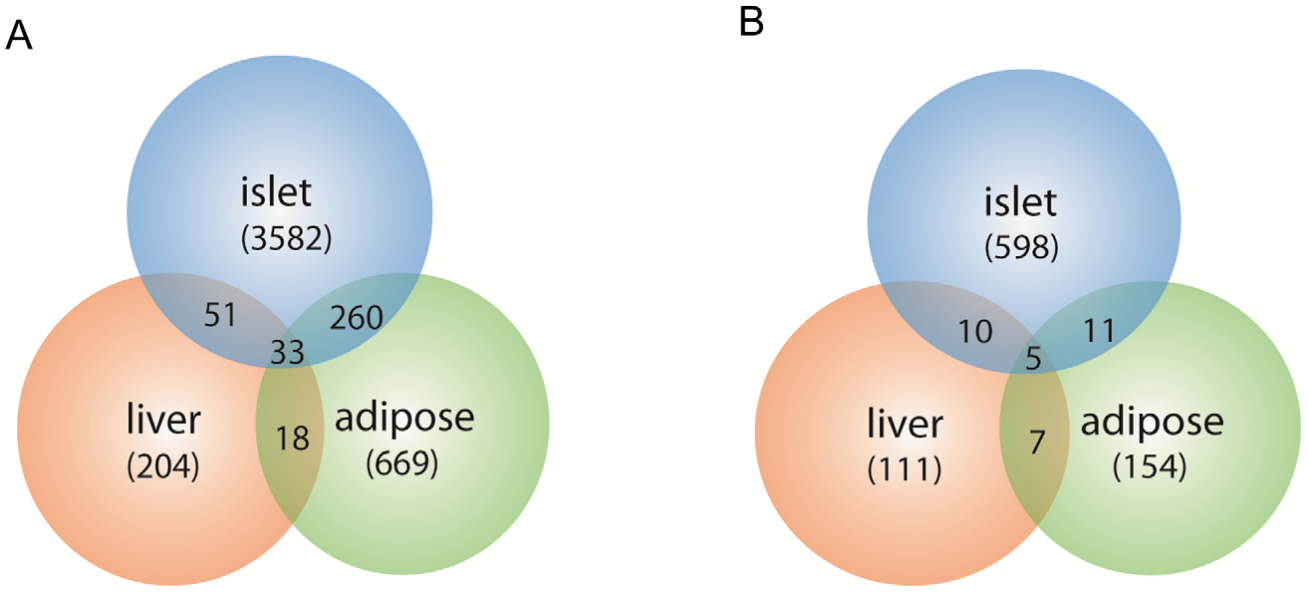
Number of gene transcripts in islet, liver, and white adipose tissue containing eQTLs that overlap with the insulin QTLs on chromosome 2 and chromosome 19. Chromosome 2 (a), chromosome 19 (b). Numbers in parentheses show tissue-specific eQTLs (before filtering out genes that were determined to be independent of the insulin trait).

**Figure 3:**
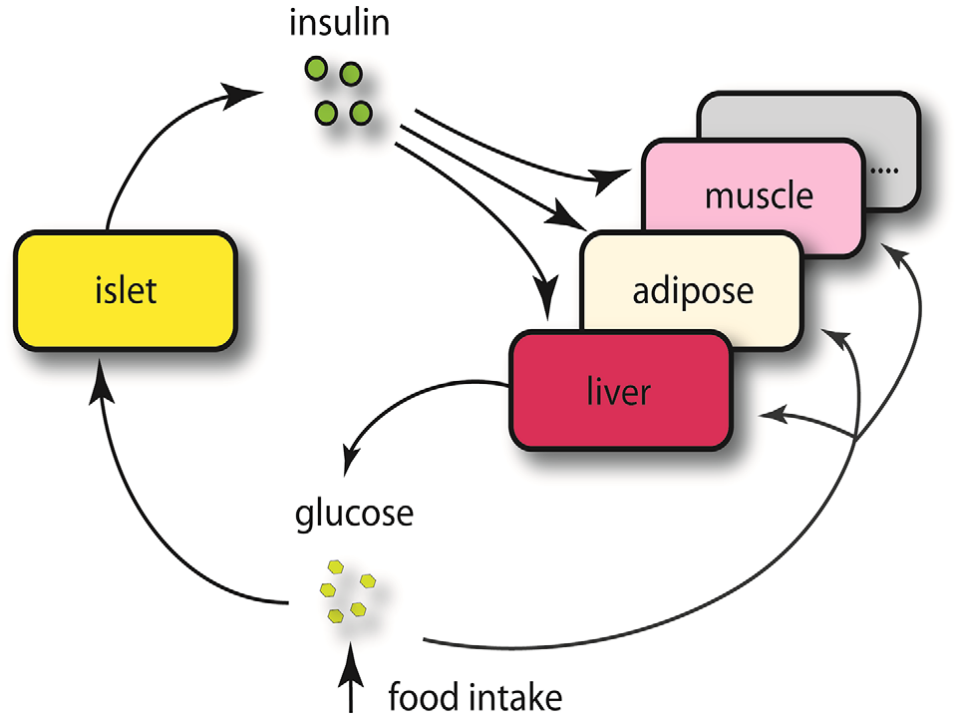
Schematics view of insulin regulation. Elevated glucose level by either food intake or liver glycogenolysis is sensed by islet and leads to insulin secretion to the bloodstream. The increased insulin stimulates peripheral tissues to absorb glucose, and as a consequence, the glucose level in the plasma would return to normal level. Since a loop is formed regarding insulin regulation, it is necessary for us to consider both casual and reactive genes to insulin.

The expression traits that overlapped with the insulin QTLs were tissue-specific, and are enriched in different GO biological pathways (Table S2). The largest number of these traits was from pancreatic islets (Table 2, Figure 2). In addition, islets contained the largest proportion of eQTLs that showed linkage to both loci on chromosomes 2 and 19, indicating that similar to traditional complex traits (e.g. insulin), gene expression is also regulated by multiple genetic loci [28]. Co-localization of gene eQTLs and plasma insulin QTLs does not imply the gene is related to plasma insulin regulation. To filter out genes that, while linked to the same QTL region as insulin QTLs, are likely independent of plasma insulin regulation (described in Methods and Text S1), we applied a genetic causality test developed by Schadt *et al.* [13] to further narrow our list of candidate regulatory genes. Given the known feedback loop (shown in Figure 3) (islets – insulin levels – peripheral tissues, and – glucose levels), genes supported as either causal (QTL <$>\scale 80% \raster="rg1"<$> gene <$>\scale 80% \raster="rg1"<$> insulin), or reactive with respect to insulin levels (QTL <$>\scale 80% \raster="rg1"<$> insulin <$>\scale 80% \raster="rg1"<$> gene) were identified for consideration as insulin regulation genes.

**Table 2:** Number of genes whose eQTL mapped to chromosome 2 and 19 for five tissues before filtering out genes that were determined to be independent of the insulin trait.

Number of Genes	Islet	Adipose	Gastroc	Liver	Hypothalamus
**eQTL on Chr 2 (FDR)**	3926 (4.0%)	980 (12.0%)	779 (15.4%)	306 (43.3%)	139 (85.5%)
**eQTL on chr 2 causal or reactive**	1703	399	30	47	4
**eQTL on Chr 19 (FDR)**	624 (12.0%)	177 (25.4%)	142 (47.8%)	133 (43.3%)	101 (52.2%)
**eQTL on chr19 causal or reactive**	516	88	11	34	9
**Overlap btw 2/19**	380	26	18	7	5
**Overlap btw filtered 2/19**	295	10	1	1	0

FDR: False Discovery Rate.

### Constructing protein–protein interaction network for genes with common eQTLs

**Figure 4:**
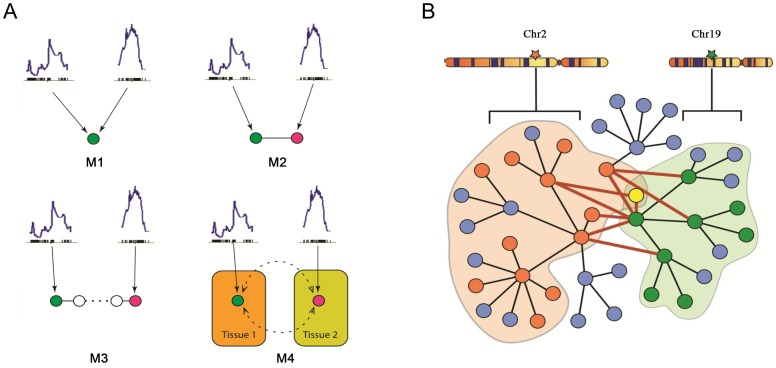
Mechanistic models for joint regulation by two loci and interactive model for the additive effects between two genetic loci that regulate plasma insulin. A) Mechanistic models for joint regulation by two loci. In model 1, two loci directly regulate same gene. In model 2, each locus regulates one gene, and the two genes have physical interaction. Model 3 is similar to model 2, but there are multiple steps between the two genes. Model 4, each locus regulates one gene in a single tissue, and the cross-tissue interaction leads to joint effect on phenotypic variation. B) Interactive model for the additive effects between two genetic loci that regulate plasma insulin. Genetic variation at chromosome 2 changes expression for some nodes in the network (orange), while variation at chromosome 19 changes expression of other nodes (green) including genes regulated by chromosome 2 (yellow node). The blue nodes represent other nodes in the global protein-protein interaction network. Genes bound by grey curves are genes sharing same eQTLs. Nodes involved in an interaction between the two sub-networks (shaded in light orange and green) are connected by bold red lines. We hypothesize these nodes would be more influenced by genetic variation at both loci than a single locus. Genes involved in these cross-group interactions may be key regulators of plasma insulin.

A model considering the two loci, on chromosomes 2 and 19, accounts for a greater part of the variation in plasma insulin than a single locus model. Several models of various degrees of complexity could explain the joint regulation of a common trait by multiple loci. As plotted in Figure 4A, the simplest case (M1) would be that the two loci directly regulate the same gene and that such a gene is responsible for modulating the trait. A slightly more complex case (M2) would be each locus regulates a different gene, which could collaborate directly through protein-protein physical interaction to influence the trait. In model M3, genes regulated by different loci interact indirectly and multiple steps exist before the perturbation signals merge on the common trait. In model M4, multiple tissues and their interactions are involved in regulating the trait. Here, we developed an approach that seeks to combine the first two models to identify those components of the network underlying insulin regulation that are modulated by the chromosome 2 and 19 genetic loci and that may be physically interacting. The more complex models is not considered here but left for future work. As shown in Figure 4B, genetic variation at a single locus results in perturbations of biological functions that are reflected in the transcripts, or nodes, linked to that locus (orange or green nodes). Genetic variation at two loci could result in a larger functional influence on nodes showing linkage to both loci (yellow nodes) or nodes interacting across the two sub-networks (nodes connected by red edges).

Here we only consider the situation in which single genetic variations are synergistic, although antagonistic relationships may certainly occur. We hypothesize that nodes mediating the interaction between the two sub-networks are critical points for integrating the effects of multiple loci. To identify and rank these critical nodes, we developed an algorithm for assessing a gene's potential for being such an integrator in each of five tissues. We first collected a mouse protein-protein interactome by combining information from various databases as previously described [20], where most interactions were experimentally derived and manually curated. We then extract tissue-specific networks by mapping genes with eQTLs overlapping insulin QTLs on chromosome 2 or 19 onto this interactome and considering only interactions across the two eQTL gene groups. Islets contain the greatest number of genes involved in a cross group interaction (listed in Table S3) between the sub-networks showing linkage to chromosomes 2 or 19 (Table 3).

**Table 3:** Genes involved in cross-group interactions.

Gene set	Islet	Adipose	Liver
**eQTL on Chr 2**	341/823	35/201	0/23
**eQTL on Chr 19**	125/225	12/42	0/19
**#Interaction**	1349 (0.73%)	119 (1.4%)	0 (0.0%)

The first number in the first two rows indicates the number of genes that are involved in cross group interactions and whose eQTLs are mapped to either chromosome 2 or 19. The second number indicates the number of genes whose eQTLs mapped to chromosome 2 or 19 and are contained in the protein interaction database that we used to construct the network. The third row shows the number of cross group interactions in each tissue and the number in the parenthesis indicates the frequency of observing such cross group interaction. Using islet as an example, we have 823 genes in the first group and 225 genes in the second group, the total possible cross group interactions are 823×225. The actual number of interactions we observed across these two groups of genes is 1,349. Therefore, the frequency is 0.73% (1349/(823×225)). By comparing the frequency, we can tell if in a particular tissue the cross-group interactions occurred at a particularly high frequency or vice versa.

**Figure 5:**
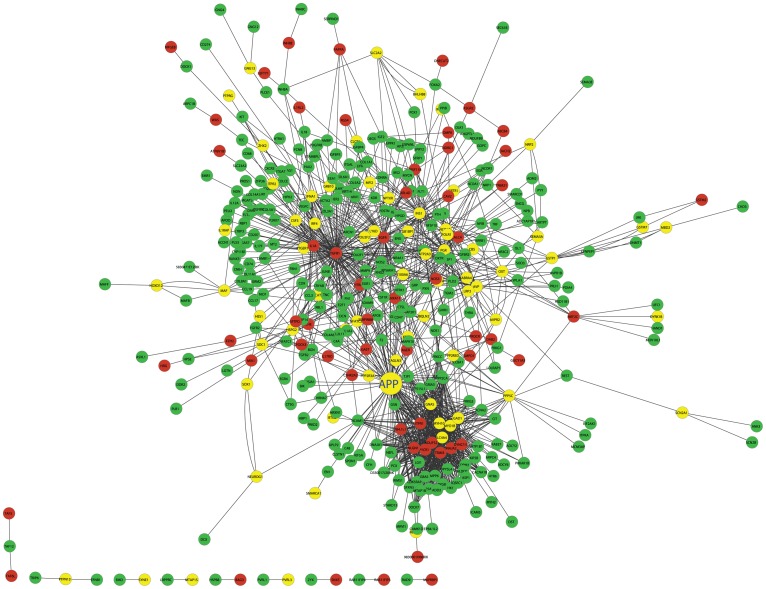
The cross eQTL group protein–protein interaction network in islets. Only those genes with eQTLs to chromosome 2 or 19 are shown. Nodes in green/red color are genes with eQTLs on chromosome 2/19; yellow color represents linkage to both loci. Figure is drawn using CytoScape [57]. A zoomable version is shown as Figure S8.

Figure 5 illustrates the islet protein-protein interaction network constructed from gene transcripts showing linkage to either chromosome 2, 19 or both loci. Genes contained within the islet network are significantly enriched for several gene ontology (GO) categories (Table S4), such as “neuron projection” ( *p* = 5.6×10^−8^), “extracellular space” (*p* = 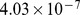), and “hormone activity” (*p* = 3.2×10^−6^). These results demonstrate that our network identifies gene sets with common biological functions and some of these functions appear to be related to insulin secretion.

### Prioritization of genes based on cross-group interactions

As shown in Figure 5, many genes are supported as being involved in cross group interactions and could conceivable play a critical role in regulating plasma insulin levels. To assess their potential in mediating cross group interactions and regulating clinical traits, we designed a novel ranking algorithm that integrates Trait, protein-protein Interaction, and gene Expression (referred to as, TIE score) to identify those genes most likely to play a critical role in insulin regulation. Instead of focusing on the property of individual genes, the TIE score incorporates an *Interaction Potential* (IP) between a protein pair. Because PPI data are not assayed in a relevant physiological context, we leverage the expression data, which is assayed in a relevant context, to weigh whether a protein interaction pair is relevant to our context of interest (i.e., interactions between diabetes-relevant tissues in the cross population). There are many post-transcriptional and post-translational modifications that may impact protein-protein interactions and their resulting functional activities. However, these modifications cannot be inferred by gene expression profiling. For a pair of proteins known to interact, we make the simplifying assumption that their protein activities and binding affinity do not change. We further assume a strong interaction potential (IP) if both genes are highly expressed in a mouse relative to the two genes in other F2 mice (since protein-protein association rates depend on protein concentrations in a simple diffusion model); if one or the other gene has relatively low expression, the IP will be weaker. We then calculate the correlation between the IP and trait, yielding a Trait – IP Correlation (TIPC). The TIPC represents the potential of an interaction (instead of individual genes) in regulating a clinical trait. Using TIPC as a weight for each interaction in the protein-protein interaction network, the TIE score is then computed for each node based on the small sub-network formed by the node and its direct neighbors (see Method for details). A node receives a high TIE score if it has numerous interactions with large TIPC values. TIE scores are context dependent so that a given gene may have different TIE scores in different tissues, given that the levels of its own expression and that of its interaction partners' may be different between tissues. In contrast to other network-based approaches [29]–[31], the TIE score enables us to identify key nodes within a network that have multiple protein interactions with neighboring nodes, where such interactions are supported as exerting a strong influence on the particular clinical trait under investigation (in our case, plasma insulin levels).

For ranking purposes, we limit our calculation to genes with 5 or more interactions and set the TIE score to zero for the rest of the genes, given genes with too few interactions may spuriously influence the TIE score and thus lead to unreliable results. We also permutated gene expression for the nodes in the network a thousand times and calculated the empirical distribution of TIE scores to assess the significance of derived TIE scores (see supplementary methods in Text S1).

Pancreatic islets had the greatest number of genes with a significant non-zero TIE score (Table 4, and listed in Table S5). Adipose tissue had many fewer genes with a non-zero TIE-score (listed in Table S6) than islets, and the other three tissues had no genes with non-zero TIE scores, suggesting that protein-protein interactions in these tissues may not be the mechanism underlying the cross-locus regulation of plasma insulin involving chromosomes 2 or 19. The top 5 ranked genes for both adipose and islet tissues are given in Table 4. Our result suggests that pancreatic islets contribute the most to variation in plasma insulin in our F2 cross between B6 and BTBR mice. Circulating levels of plasma triglyceride, an indicator of insulin resistance, showed no significant genetic linkage (Figure S2), suggesting that factors controlling insulin resistance may be distinct from that controlling plasma insulin in our B6XBTBR-ob/ob F2 cross.

**Table 4:** Top 5 genes in adipose and islet tissues ranked by TIE scores and their gene expression-insulin correlation.

Rank	Gene sym	# Interactions	TIE score	P-value	Corr. Ins
*adipose*					
1	*Cdkn1a*	14	0.23	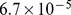	0.29
2	*Aldoa*	18	0.052	1	0.46
3	*Src*	22	0.031	1	−0.22
4	*Tpi1*	16	0.027	1	0.51
5	*Pcx*	16	0.016	1	0.30
*islet*					
1	*App*	102	0.78	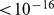	−0.62
2	*Gria3*	15	0.52	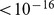	−0.57
3	*Grb10*	7	0.52	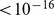	−0.52
4	*Calca*	13	0.49	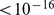	−0.39
5	*Ins1*	47	0.49	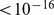	0.55

### Top ranked genes in adipose tissue and islets

*Cdkn1a* (cyclin-dependent kinase inhibitor 1 A), the top ranking gene in adipose tissue, is a potent cyclin-dependent kinase inhibitor ( p-value = 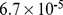). The protein binds to and inhibits the activity of cyclin-*Cdk2* or –*Cdk4* complexes, and thus functions as an inhibitor of cell cycle progression at G_1_. The expression of this gene is tightly controlled by the tumor suppressor protein p53, in response to a variety of stress stimuli [32]. Previous reports demonstrate that p53 expression in adipose tissue is crucially involved in the development of insulin resistance [33]. The p21 KO mouse (*Cdkn1a*−/−) showed 90% increase in fat pad weights, 70% increase of adipocyte numbers, and insulin resistance [34].

Compared to the top scores in adipose tissue, the top scores for genes in the islet network are much higher (Table 4), suggesting a greater number of genes in islet make a larger contribution to insulin variation. The top ranked gene in the islet network is *App* (Amyloid β Precursor Protein). Successive proteolytic processing of APP by β- and γ- secretase enzymes generates the amyloid-β peptide, a primary component of amyloid plaques, which are thought to be central to the etiology of Alzheimer's disease (AD) [35]. Although this gene has been heavily studied by AD researchers, its relevance in type 2 diabetes is much less known. In addition to *App*, several other genes with high TIE scores could potentially be involved in regulating plasma insulin. Wang *et al.* showed that peripheral-tissue-specific knockout of *Grb10* results in enhanced insulin sensitivity *in vivo* [36] that could be due to the loss of *Grb10*-mediated degradation of the insulin receptor [37]. More recently, disrupting *Grb10* is shown to increase pancreatic beta cell mass and reduce beta cell apoptosis in mice [38]. The enrichment of genes previously shown to participate in the regulation of circulating insulin in the top islet gene list, such as *Grb10* and insulin *Ins1*, supports our use of TIE to identify novel regulators of insulin.

### App negatively regulates insulin secretion from pancreatic islets

The expression of *App* in islets strongly negatively correlates with plasma insulin levels in the F2 cross (Pearson correlation R = −0.68, p-value≪0.01, Figure S3). We have previously characterized the difference in diabetes susceptibility between the two parental mouse strains [24]. At 10 weeks of age, BTBR-ob/ob mice are diabetic, whereas B6-ob/ob remain euglycemic. BTBR-ob/ob mice have lower plasma insulin and a higher level of *App* in islets than B6-ob/ob mice (difference in *App* expression p-value∼0.05, Figure S4), which is consistent with the negative correlation that we observed in the F2 cross. The distributions of the islet *App* expression levels as a function of genotype at the Chr. 2 and 19 loci (Figure S5) indicate that F2 mice with BTBR genotypes at the two loci have higher islet *App* expression, consistent with *App* gene expression negatively correlating with plasma insulin levels.

Previous work has shown that compared to wild-type mice, whole-body *App* knockout mice (*App^−/−^*) have reduced plasma glucose and elevated insulin secretion in response to an intravenous glucose injection [39]. Given that *App* is expressed in multiple tissues, including the brain where it may regulate neurogenesis [40], we sought to determine if the changes observed in *App^−/−^* mice reflect direct or indirect effects of *App* on islet function and/or health.

**Figure 6:**
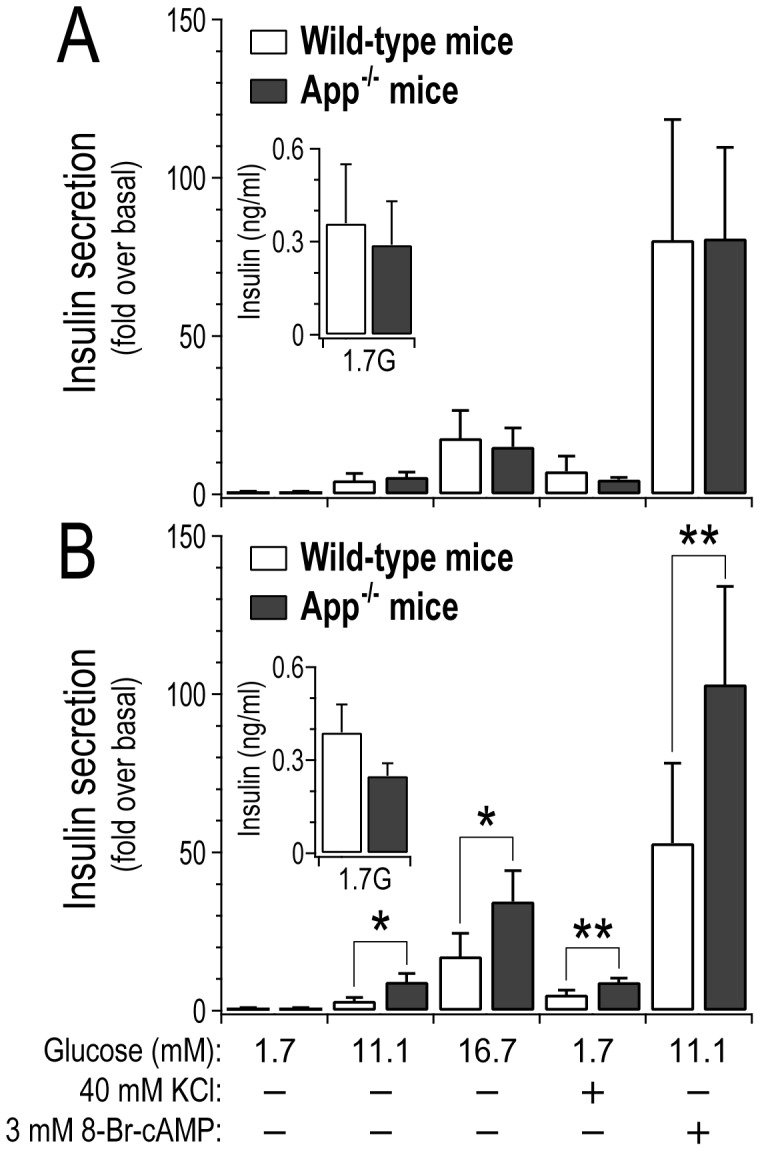
Loss of *App* leads to increased insulin secretion from islets in older mice. Islets were collected from 10 week old (A) or 17 week old (B) male wild-type or whole-body *App^−/−^* mice. Insulin secretion was stimulated by varying glucose concentrations, a depolarizing concentration of KCl, or 8-Br-cAMP, a membrane-permeant analogue of cAMP. Data expressed relative to secretion observed in response to 1.7 mM glucose, 4.7 KCl and no cAMP (basal). *, *p*<0.05; **, *p*<0.01 for *App^−/−^* versus wild-type mice. Inserts show average basal insulin secretion (ng/ml) from 3 islets per genotype in response to 1.7 mM glucose (n = 3 for each experimental group).

To assess whether the loss of *App* has a direct impact on islet function, we monitored insulin secretion *ex vivo* from pancreatic islets collected from either wild-type or *App^−/−^* mice (Figure 6). At 10 weeks of age, insulin secretion from wild-type versus *App^−/−^* mice was not different (Figure 6A). However, at 17 weeks of age insulin secretion was elevated ∼2–3-fold from *App^−/−^* islets in response to glucose (p-value<0.05), a depolarizing concentration of KCl (p-value<0.01), or a membrane-permeant analogue of cAMP (*p*<0.01) (Figure 6B). The amount of insulin per islet (insulin content) was not significantly different between wild-type and *App^−/−^* mice (Figure S6). Further, insulin secretion in response to basal glucose concentration (1.7 mM) at either 10 or 17 weeks of age was not significantly different between wild-type and *App^−/−^* mice (Figure 6, inserts). These results suggest that *App* directly functions as a negative regulator of insulin secretion in islets, and this only occurs in older mice.

## Discussion

We developed a novel network model that integrates genetic, transcription and protein-protein interaction information to pinpoint *App* as a key insulin regulatory molecule in pancreatic islet tissue. The computational model we developed has several unique features.

Instead of pursuing *cis*-regulating genetic factors, it focused on networks of genes that were *trans*-regulated. The goal was not to identify the genetic factors whose variation at DNA level would lead to changes in circulating insulin. Instead, the model identifies networks of genes showing transcriptional changes as result of variation in the genetic factors. This is based on the assumption that the disease phenotype is at least partially mediated by these transcriptional changes. Genes identified by this approach could also have a more direct link to the disease phenotype compared to the upstream genetic factors. The model also simultaneously considered multiple loci, which enabled the study of the interactions between *trans*-regulated gene modules. As it is extremely common for complex disease phenotype traits to map to multiple loci, it is clear that we need models considering the joint effects of multiple loci. Ideally such models should not only be meaningful in the mathematical terms, but also provide biological insight to the possible mechanisms. Although the linear regression model indicated a joint regulation of the insulin trait, it did not generate any hypotheses on how the joint regulation occurred biologically.

Compared to other network models, such as co-expression network [41], ARACNE [9], and Bayesian network [22], [42], which focus on grouping co-expression of individual genes, our method focuses on dissecting potential mechanisms of integrating information from multiple co-expression modules. By considering the protein-protein interactions across the two groups of genes, it is possible to actually identify potential molecular mechanisms involved in joint regulation. Although currently the protein-protein interaction dataset we compiled may be rather incomplete, hundreds of genes were connected by these interactions. This makes prioritizing genes for experimental validation a more important task compared to finding out what could have been missed due to incomplete protein interaction information. To prioritize the key nodes in the disease network, we developed the novel scoring system in the context of the protein interaction network. As we posit that proteins their function by interacting with their neighbors in the network, the TIE score gives a weighted estimation on how strongly the intensity of these interactions correlates with the phenotype. A gene with high TIE score suggests that the intensity of its interactions strongly correlates with the phenotype based on large numbers of interactions. Therefore, the gene is likely to regulate the trait. By integrating genetic, gene expression, and phenotypic trait information, the ranking algorithm identified biologically meaningful candidate insulin regulators.

A previous publication has shown that, compared to wild-type mice, whole-body *App* knockout mice (*App^−/−^*) have elevated insulin secretion in response to an intravenous glucose injection [39]. A recent study of the cross of *App* transgenic mice and T2D predisposition mice shows that increased Aβ production impairs insulin signaling and accelerates insulin resistance [43]. To our knowledge, however, no other studies have demonstrated a direct effect of APP on islet function. Given that *App* is highly expressed in pancreatic islets [44]–[45], we sought to determine if the changes observed in *App^−/−^* mice reflect direct or indirect effects of *App* on islet function. Our measurements of glucose stimulated insulin secretion in isolated islets from *App* KO mice confirms our network analysis and is also consistent with the causality test [13] which also indicates *App* as a causal gene in pancreatic islet tissue (Figure S7). The model demonstrates that *App* is under the regulation of multiple genetic loci, and may function as an integrator for these perturbation signals, mediating interactions between two distinct gene sets that share a common genetic architecture with plasma insulin.

We have previously shown that Lep^ob/ob^ mutation exposes a strain-dependent difference in diabetes susceptibility between BTBR and B6 mice [25]. In the current study we exploited this difference and used it as a “sensitized screen” to genetically map genes and diabetes-related clinical traits that may underlie this difference. This approach allowed us to identify *App* as a key negative regulator of insulin secretion from pancreatic islets. In this study, we compared wild-type and *App−/−* mice to test for a direct role of *App* in insulin secretion in mice not expressing the Lep^ob/ob^ mutation. In these studies, the loss of *App* resulted in enhanced insulin secretion, consistent with the strong negative relationship between islet *App* and circulating insulin across the F2 samples. These results suggest that while leptin deficiency was critical in revealing the islet network involving *App* and circulating insulin, it was not required to demonstrate the direct role of *App* in insulin secretion.

Our results, which demonstrate a difference in insulin secretion between islets collected from wild-type and those collected from *App^−/−^* mice at 17 weeks, but not 10 weeks of age, implies an age-dependence for the role that *App* plays in the islet. However, studies in mouse [46] and human islets [47] have not reported an age-dependent change in *App* expression. It is possible that proteolytic processing of *App* mediated by the beta- and gamma-secretase enzymes, or other forms of post-translational modification, are necessary for *App* to regulate insulin secretion.

Mouse and rat beta cells are more sensitive to oxidative stress than human beta cells [48]–[49], due to the relatively higher expression of antioxidant enzymes in human beta cells [47], [50]. We showed that the sub-network regulating plasma insulin level variation (Figure 5) is enriched for GO categories “neuron projection” (*p* = 5.6×10^−8^), “extracellular space” (*p* = 4.03×10^−7^), and “hormone activity” (*p* = 3.2×10^−6^). Genes involved in the stress response process are not enriched in the subnetwork. Recent RNAseq data [47] suggests that *APP* robustly expresses in human islet cells. In addition, it has been shown that aggregated amyloid-β peptide as well as other proteins have been detected at higher levels in pancreatic islets of T2D patients comparing to healthy control people [51]. These suggest that the subnetwork and key regulators in mouse islet we identified in the F2 cross are expected to be relevant in human islets. Our findings support the hypothesis that APP contributes to the common pathogenesis of AD and T2D [52].

For the future development, (1) a generalized multi-way interaction model is needed to capture complex interaction networks underlying complex traits such as plasma insulin; (2) additional experiments are needed to systematically validate candidate genes (such as genes in Table S5 and genes connected to *App* in Figure 5) for their roles in affecting β-cell function which in turn affect insulin production and insulin secretion; (3) the molecular mechanism of age-dependent *App* regulating insulin secretion is warrant further study.

In conclusion, using an integrative analysis of gene expression, genotypes, and phenotypic traits of the B6xBTBR *ob/ob* F2 cross, we showed that plasma insulin is modulated by the variation of multiple genetic factors, presumably through expression changes of hundreds of genes in multiple tissues. Our approach focused on revealing the underlying disease network across loci and tissues. The model predicted that *App* acts in pancreatic islets to affect plasma insulin. This prediction was tested in isolated islets where the knockout of *App* was associated with increased insulin secretion. Considering *App* is known for Alzheimer's disease development and a strong association between T2D and AD, our findings point to a potential mechanism through which these two diseases are linked.

## Materials and Methods

### Animal husbandry, tissue collection, and molecular profiling

All animal studies were conducted at the University of Wisconsin in the Biochemistry Department in accordance with NIH guidelines and the University of Wisconsin Research Animals Resource Center. *App−/−* mice were purchased from the Jackson Labs (stock number 004133). C57BL/6J (B6) *ob/+* male and BTBR *ob/+* female mice were bred to obtain F1 *ob/ob* mice [24]. Leptin deficiency causes infertility [53]–[54]. To restore fertility to F1 ob/ob mice, at approximately 4 weeks of age the F1 *ob/ob* male and female mice each received subcutaneous transplants of white-adipose tissue from lean (leptin-competent) litter mate donor mice, resulting in the restoration of fertility in >90% of the F1 ob/ob mice. The F1 *ob/ob* mice were then bred to produce a panel of ∼550 F2 *ob/ob* mice. At 10 weeks of age, the F2 ob/ob mice were sacrificed and tissues collected (islet, white adipose, liver, gastroc muscle, and hypothalamus). Gene expression was profiled on an Agilent custom murine gene expression microarray consisting of 4,732 control probes and 39,558 non-control oligonucleotides extracted from mouse Unigene clusters and combined with RefSeq sequences and RIKEN full-length cDNA clones. All F2 mice were genotyped with the Affymetrix 5 K SNP array, which identified ∼2,000 SNPs that were polymorphic between B6 and BTBR mice that spread uniformly across genome. Various clinical traits were measured for each mouse just prior to sacrifice. See Supplementary materials (Text S1) for additional description of methods.

### Isolation of pancreatic islets

Intact pancreatic islets were isolated from mice using a collagenase digestion procedure [55]. Briefly, the mice were sacrificed and the pancreases immediately inflated with 5 ml Hanks Buffered Salt Solution (HBSS) supplemented with 0.02% BSA and collagenase (0.5 mg/ml). After inflation, the pancreata were carefully dissected from the mice, placed in 25 ml of HBSS/BSA/collagenase, and incubated for 16 min at 37°C, with intermittent agitation. A ficoll gradient was used to partially purify islets from the digested pancreata, and further purified by hand-picking the islets viewed under a stereo-microscope. Media used for isolation and insulin secretion studies was a Krebs-Ringer Bicarbonate Buffer (KRB) containing (in mM): 118.41 NaCl, 4.69 KCl, 2.52 CaCl_2_, 1.18 MgSO_4_, 1.18 KH_2_PO_4_, 25 NaHCO_3_, and 5 HEPES.

### Plasma insulin measurement

For the measurement of plasma insulin, all mice were fasted beginning at 8 AM, by transfer to a clean cage and provided water *ad libitum*. Approximately 4 hours later, ∼0.1 ml of blood was collected via retro-orbital draw, transferred to a tube containing 3 µl of 0.5 mM EDTA as the anti-coagulant, and then centrifuged (5 mins, 10,000×*g*, 4°C) to isolate plasma. The level of plasma insulin was measured as described previously [24]. Briefly, high-binding plates (Corning) were coated overnight with 3 µg/mL of an anti-insulin antibody (D6C4, Research Diagnostics), blocked with PBS containing 4% RIA-grade BSA (Sigma) for 1 h and then incubated for 1 h with insulin standards (Linco Research, 0.1–10 ng/mL) or 25 µl whole plasma. An anti-proinsulin antibody (1 µg/ml of D3E7-BT, Research Diagnostics) was added and incubated for an additional hour. After extensive washing (50 mM Tris, 0.2% Tween-20, pH 8.0), 1 µg/mL of streptavidin-HRP (Pierce) in PBS/0.1% BSA was added and incubated for 30 min. Following additional washes, 16 µmol/ml of *o*-phenylenediamine (Sigma), dissolved in citrate buffer (0.1 M citrate-phosphate, 0.03% H_2_O_2_ at pH 5.0), was added and incubated for 30 min; 0.18 M sulfuric acid was used to quench the reaction. Absorbance at 492 nm was determined by a plate reader (Ultra 384 TECAN). Insulin contents in plasma were calculated by comparison to known standards.

### Insulin secretion

Three islets of equivalent size were placed in 12×75-mm glass tubes, where the bottom of the tube was formed by a 62-µm mesh (Elko Filtering Co.). The 12×75-mm tubes were transferred to 16×100-mm tubes containing 1 ml of KRB with 1.7 mM glucose and 0.5% BSA and pre-incubated at 37°C for 45 min. Following the pre-incubation, the 12×75-mm tubes were transferred to a fresh 16×100-mm tube containing 1 ml KRB supplemented with 1.7, 11.1 or 16.7 mM glucose, with or without additional KCl or 8-Br-cAMP as indicated. For studies where 40 mM KCl was added to the secretion medium, NaCl was reduced to 78.41 mM to maintain osmolarity. Following a 45-min incubation period at 37°C, the 12×75-mm tubes were transferred to a fresh tube containing 1 ml of HCl-ethanol-water (1∶50∶14) to extract cellular insulin from the islets. The incubation media was collected and frozen for insulin determination by ELISA.

### cQTL/eQTL mapping and testing for causal versus reactive genes

Insulin trait cQTL and gene eQTL mapping were performed using scanone function in R package R/qtl [56]. The causality test was described previously [13] and a Bayesian network version was used to conduct the test.

### Network construction and TIE scores for ranking genes involved in cross-group interaction

The global mouse protein-protein interaction network was collected as described previously [20]. For two interacting proteins 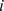 and 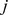, we define an *Interaction Potential* (IP) for the protein pair in an individual mouse 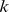 as
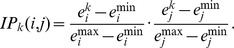


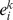
 is the gene 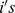 expression in mouse 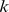, so is 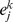 for gene 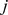. 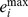 and 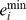 are the maximum and minimum tissue-specific expression observed across the entire F2 panel for gene 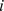. The calculation assumes the variation of protein abundance is approximated by its gene expression, and IP is proportional to the relative levels of the two proteins. Thus, a reduction in gene expression would lead to reduced protein-protein interaction for a given pair and vice versa. The predictive power of 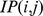 for a specific trait value (such as plasma insulin in our case) can be calculated as the Trait Interaction Potential Correlation (TIPC). We define 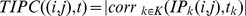, where 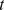 is the trait in consideration, 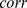 is the function for calculating the correlation coefficient, and 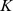 is the union of all the mice. We consider both network topology and TIPC scores to rank genes. Assume protein 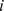 interacts with a set of proteins 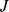, the TIE score 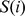 is computed as
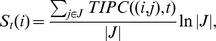
where 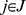 and 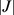 is the number of proteins contained in 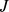. Gene with a high TIE score indicates it has large number of interactions and for its direct neighbors, the average correlation between interaction potential and trait is also high.

## Supporting Information

Figure S1 The interaction between chromosome 2 and 19 with respect to insulin level. The marker was selected based on insulin QTL mapping where the LOD score was maximized. Using linear regression modeling, the locus on chromosomes 2 and 19 explains 10.6% and 8.4% of the variation of plasma insulin, respectively; a model considering both loci jointly explains 16.8% of the variance. By comparing the distribution of plasma insulin of mice with genotype (0,2), (2,0) and (2,2), it is clear that mice with genotype (2,2) have highest plasma insulin.
(TIF)

Figure S2 Blood triglyceride, an indicator of insulin resist, is not under strong genetic control in this F2 cross as indicated by its QTL map (green curve).
(TIF)

Figure S3 The correlation between insulin and *App* gene expression in five tissues. We observe a strong anti-correlation only in pancreatic islet tissue.
(TIF)

Figure S4 The expression of *App* gene in B6 ob mice is lower than in BTBR ob mice at 10th week. Y-axis value (mlratio) is log10(ratio) where the ratio is between the expression intensity of a particular sample versus the pooled intensity of all samples.
(TIF)

Figure S5 The gene expression of *App* in F2 cross distribution in different groups based on chr2 and chr19 genotypes.
(TIF)

Figure S6 Content of insulin is not significantly different in either wild type and APP KO mice at age of both 10^th^ (A) and 17^th^ (B) week.
(TIF)

Figure S7 Conditioning on islet *App* expression, the plasma insulin QTL is no longer significant on chromosome 2 and 19. This supports that the regulation of genetic factors on chromosome 2 and 19 on insulin is mediated by *App*.
(TIF)

Figure S8 The cross eQTL group protein-protein interaction network in islets. This is the same as Figure 5, but a zoomable version.
(PDF)

Table S1 Genes located at insulin QTL loci. (a) Genes located on chromosome 2 insulin QTL region. (b) Genes located on chromosome 19 insulin QTL region.
(XLSX)

Table S2 Causal and reactive genes function enrichment based on DAVID tool.
(XLSX)

Table S3 Cross-group protein interaction list in pancreatic islet tissue as plotted in Figure 5.
(TXT)

Table S4 Gene enrichment of the TIE hits in islet network.
(XLSX)

Table S5 Ranks of genes with TIE scores in pancreatic islet.
(XLSX)

Table S6 Ranks of genes with TIE scores in adipose tissue.
(XLSX)

Text S1 Supplementary Methods: Generation of B6×BTBR cross F_2_ Mice and genotyping and gene expression data. Data analysis on eQTL mapping. Construct protein network and prioritize genes.
(DOCX)
