# Supplementary figures and images for: Integrative Analysis of a Cross-Loci Regulation Network Identifies App as a Gene Regulating Insulin Secretion from Pancreatic Islets
Source: PLoS Genet. 2012 Dec 6;8(12):e1003107. doi: 10.1371/journal.pgen.1003107 (PMC3516550; doi:10.1371/journal.pgen.1003107)

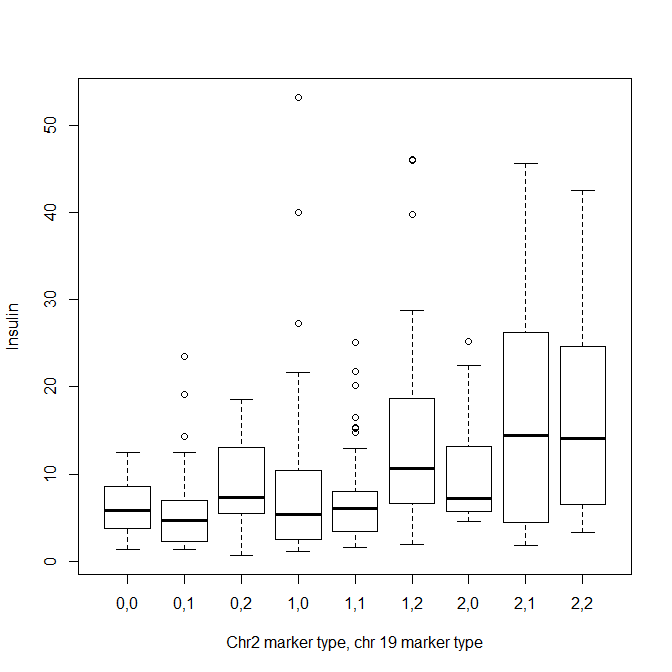

Supplement: Figure S1 — The interaction between chromosome 2 and 19 with respect to insulin level. The marker was selected based on insulin QTL mapping where the LOD score was maximized. Using linear regression modeling, the locus on chromosomes 2 and 19 explains 10.6% and 8.4% of the variation of plasma insulin, respectively; a model considering both loci jointly explains 16.8% of the variance. By comparing the distribution of plasma insulin of mice with genotype (0,2), (2,0) and (2,2), it is clear that mice with genotype (2,2) have highest plasma insulin. (TIF) [file pgen.1003107.s001.tif]

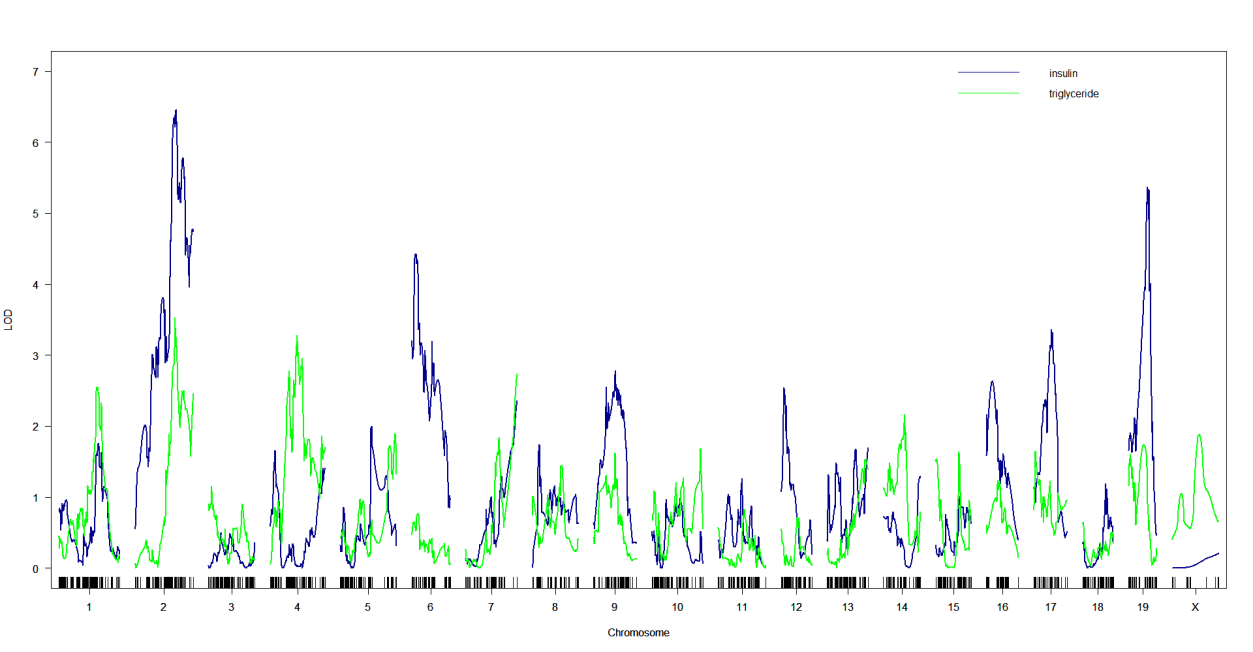

Supplement: Figure S2 — Blood triglyceride, an indicator of insulin resist, is not under strong genetic control in this F2 cross as indicated by its QTL map (green curve). (TIF) [file pgen.1003107.s002.tif]

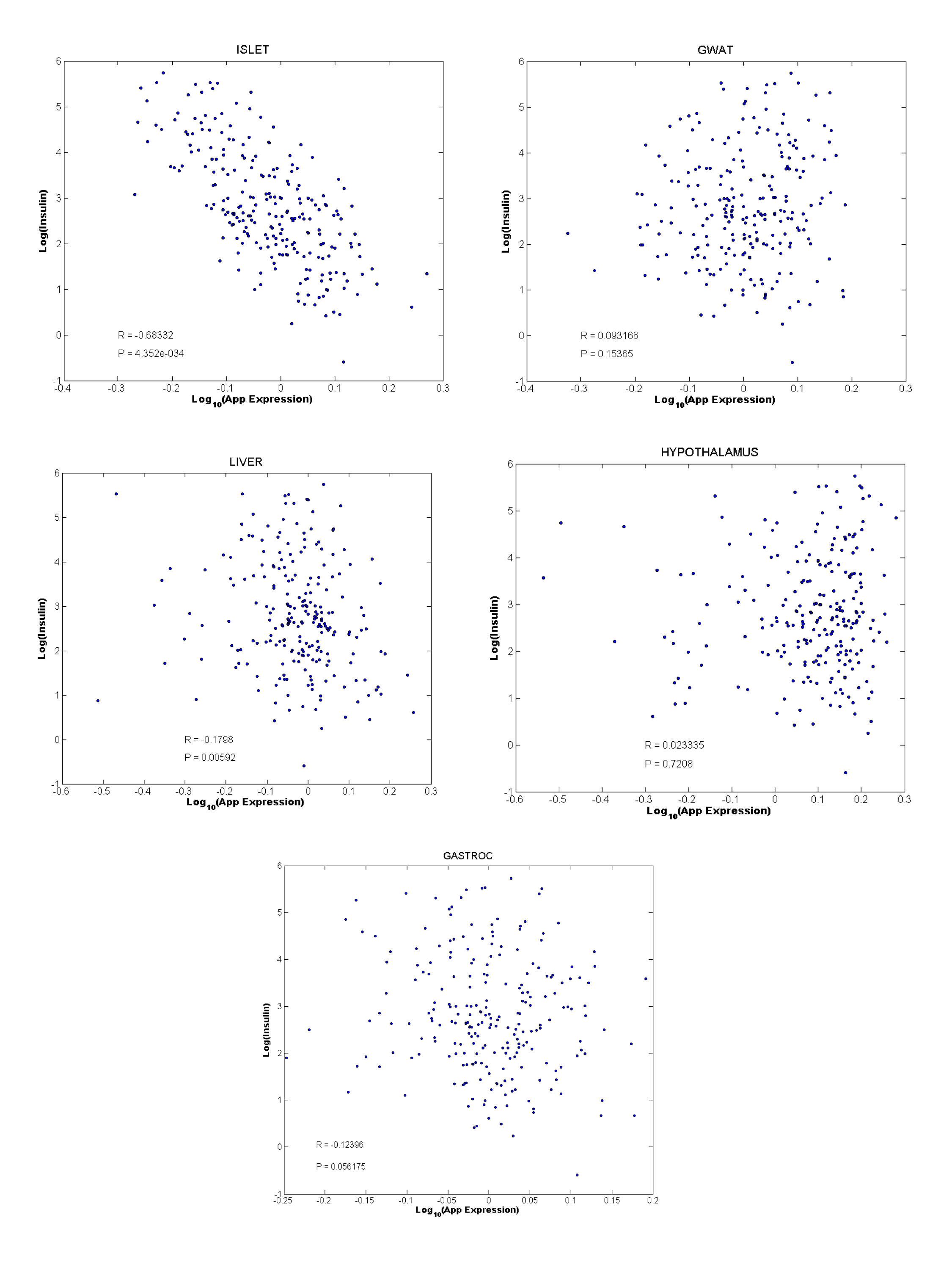

Supplement: Figure S3 — The correlation between insulin and App gene expression in five tissues. We observe a strong anti-correlation only in pancreatic islet tissue. (TIF) [file pgen.1003107.s003.tif]

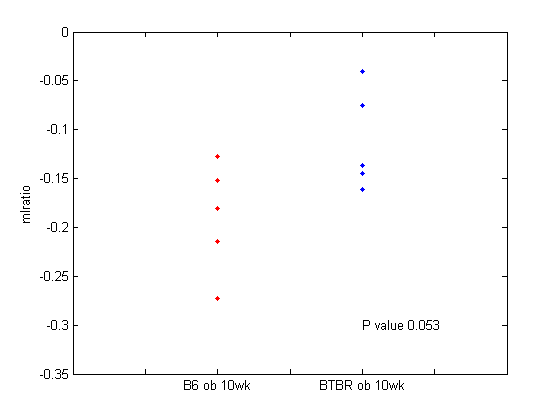

Supplement: Figure S4 — The expression of App gene in B6 ob mice is lower than in BTBR ob mice at 10th week. Y-axis value (mlratio) is log10(ratio) where the ratio is between the expression intensity of a particular sample versus the pooled intensity of all samples. (TIF) [file pgen.1003107.s004.tif]

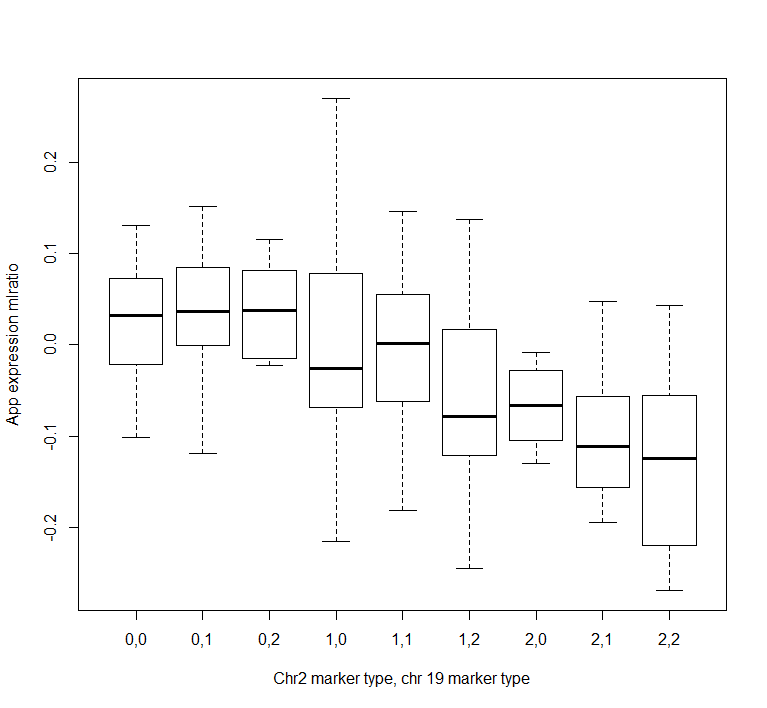

Supplement: Figure S5 — The gene expression of App in F2 cross distribution in different groups based on chr2 and chr19 genotypes. (TIF) [file pgen.1003107.s005.tif]

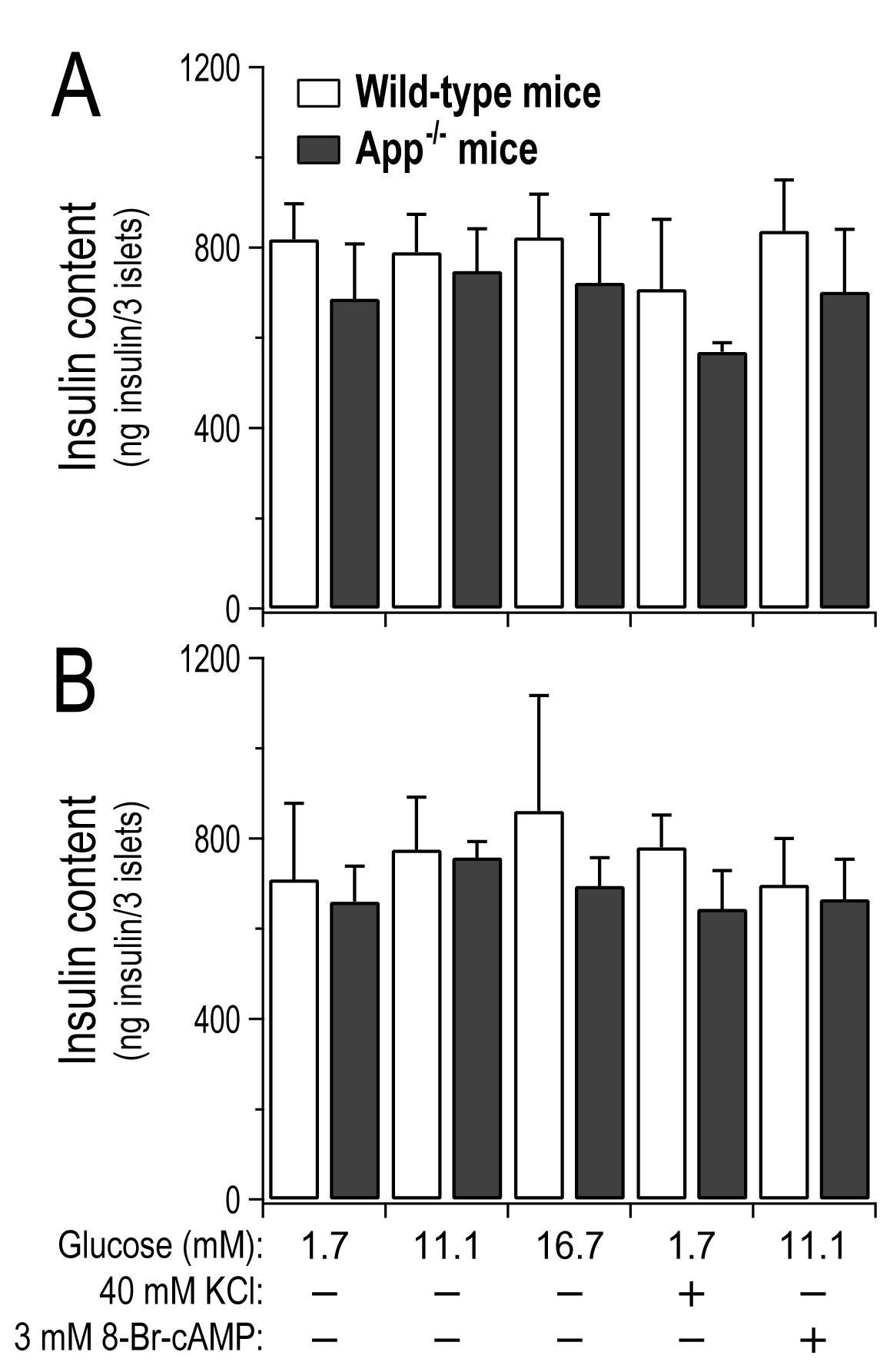

Supplement: Figure S6 — Content of insulin is not significantly different in either wild type and APP KO mice at age of both 10th (A) and 17th (B) week. (TIF) [file pgen.1003107.s006.tif]

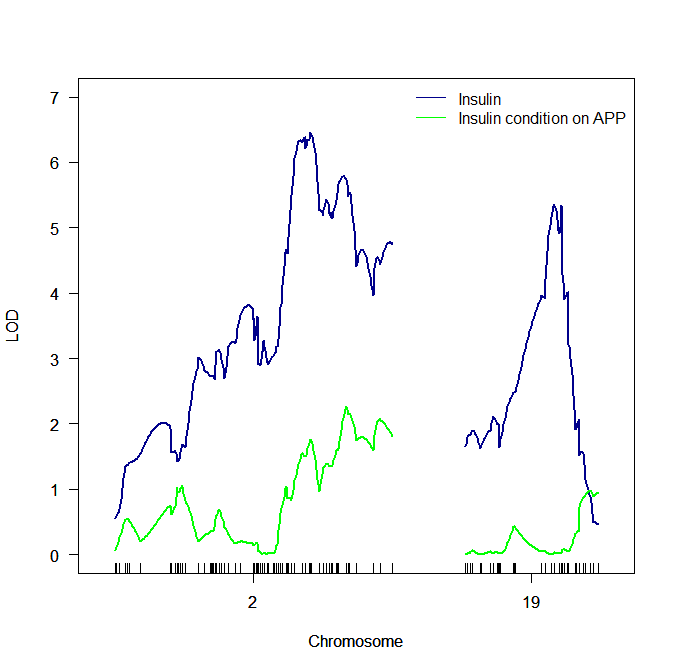

Supplement: Figure S7 — Conditioning on islet App expression, the plasma insulin QTL is no longer significant on chromosome 2 and 19. This supports that the regulation of genetic factors on chromosome 2 and 19 on insulin is mediated by App. (TIF) [file pgen.1003107.s007.tif]

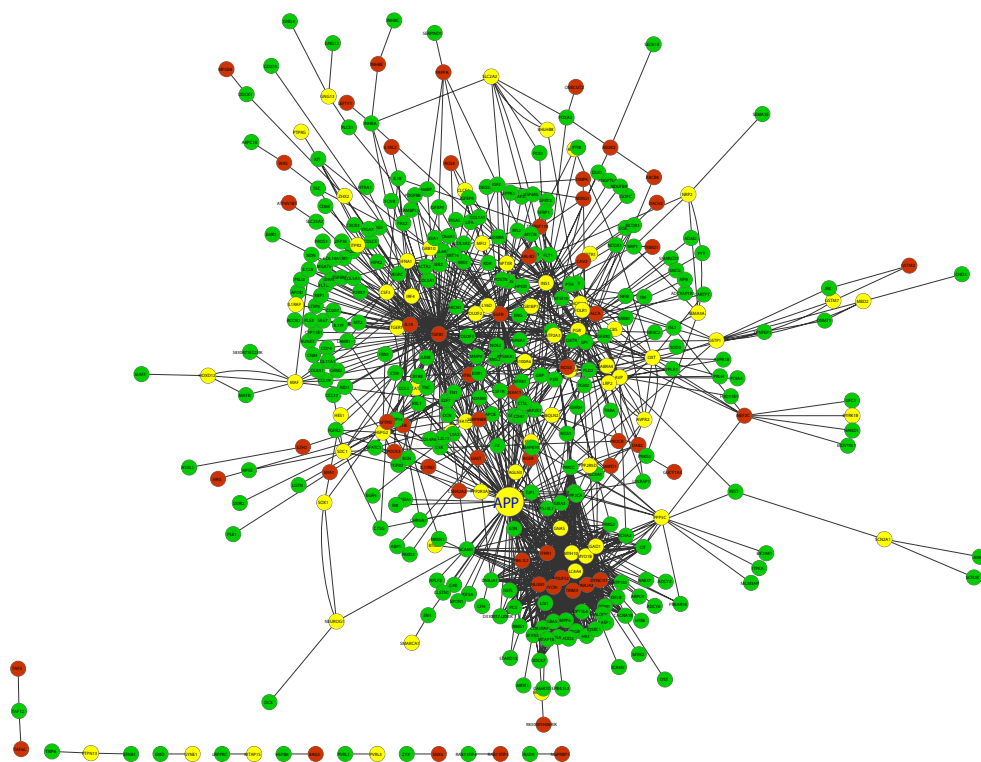

Supplement: Figure S8 — The cross eQTL group protein-protein interaction network in islets. This is the same as Figure 5, but a zoomable version. (PDF) [file pgen.1003107.s008.pdf]
