# Supplementary material for: Integrative Analysis of a Cross-Loci Regulation Network Identifies App as a Gene Regulating Insulin Secretion from Pancreatic Islets
Source: PLoS Genet. 2012 Dec 6;8(12):e1003107. doi: 10.1371/journal.pgen.1003107 (PMC3516550; doi:10.1371/journal.pgen.1003107)
Supplement: Text S1 — Supplementary Methods: Generation of B6×BTBR cross F2 Mice and genotyping and gene expression data. Data analysis on eQTL mapping. Construct protein network and prioritize genes. (DOCX) [file pgen.1003107.s015.docx]

Text S1: supplementary materials for

**Integrative Analysis of a Cross-loci Regulation Network Identifies *App* as a Gene Regulating Insulin Secretion from Pancreatic Islets**

**Zhidong Tu^1,2*^, Mark P. Keller^3*^, Chunsheng Zhang^4^, Mary E Rabaglia^3^ , Danielle M. Greenawalt^4^, Xia Yang^5^, I-Ming Wang^6^, Hongyue Dai^4^, Matthew D. Bruss^3^, Pek Y. Lum^7¶^, Yun-Ping Zhou^6^, Daniel M. Kemp^6^,** **Christina Kendziorski^8^, Brian S. Yandell^9^, Alan D. Attie^3^, Eric E. Schadt^1,2,10,11^, Jun Zhu^1,2,10§^**

**^1^** Institute of Genomics and Multiscale Biology, Mount Sinai School of Medicine, New York, NY 10029-6574, USA

**^2^** Department of Genetics and Genomic Sciences, Mount Sinai School of Medicine, New York, NY 10029-6574, USA

**^3^** Department of Biochemistry, University of Wisconsin-Madison, Madison, WI 53706, USA

**^4^** Merck Research Laboratories, Boston, MA 02115, USA

**^5^** Department of Integrative Biology and Physiology, UCLA, Los Angeles, CA 90095, USA

**^6^** Merck Research Laboratories, Rahway, NJ 07065, USA

**^7^** Genetics, Rosetta Inpharmatics LLC, a wholly owned subsidiary of Merck & Co., Inc., Seattle, WA 98109, USA

**^8^** Department of Biostatistics and Medical Informatics, University of Wisconsin-Madison, Madison, WI 53706, USA

**^9^** Department of Statistics, University of Wisconsin-Madison, Madison, WI 53706, USA

**^10^** Graduate School of Biological Sciences, Mount Sinai School of Medicine, New York, NY 10029-6574, USA

**^11^** Pacific Biosciences, 1505 Adams Dr., Melon Park, CA 94025, USA

**^¶^** Current address: Ayasdi Inc., Palo Alto, CA, 94301, USA

**^*^** These authors contributed equally to this work

**^§^** Address correspondence to:

Dr. Jun Zhu

Mount Sinai School of Medicine

One Gustave L. Levy Place
New York, NY 10029-6574, USA

Email: jun.zhu@mssm.edu

Running Title: Integrative Analysis of a Complex Disease Network

1. **Supplementary Methods**

***1.1 Generation of B6×BTBR cross F_2_ Mice and genotyping and gene expression data***

The construction of F_2_ mice is described in the Method section in the main text and also by Zhong et al. ([Zhong, Beaulaurier et al. 2010](#_ENREF_8)). Briefly, F2 mice were generated in a cross of two inbred strains, both containing the *ob* mutation at the leptin locus: C57BL/6 *ob/ob* and BTBR *ob/ob* (referred to as the B6×BTBR cross) ([Stoehr, Nadler et al. 2000](#_ENREF_6)). All F_2_ animals were maintained on a chow diet for ten weeks and were clinically characterized with respect to obesity- and diabetes-related traits at the timepoints of four, six, eight and ten weeks. Further details regarding the plasma glucose and insulin measurements, as well as islet isolation procedures, can be found in Keller et al. ([Keller, Choi et al. 2008](#_ENREF_4)). RNA was prepared using the same methods as described previously ([Keller, Choi et al. 2008](#_ENREF_4)) and hybridized to Agilent custom murine gene expression microarrays for profiling. Genotyping of mouse was performed using affymetrix 5k SNP array following manufacture’s protocol.

***1.2 Data analysis on eQTL mapping***

Insulin QTL and gene expression eQTL analyses were performed using scanone function in R package R/qtl ([Broman, Wu et al. 2003](#_ENREF_2)) using default parameters. For insulin QTL, drop of 1.5 LOD score was used to define QTL regions. For eQTL mapping, We used QTL with pleiotropic effects on expression and metabolic traits were identified using a multivariate likelihood test ([Jiang and Zeng 1995](#_ENREF_3)). We consider a gene co-maps to the same QTL if its maximum LOD on chromosome 2 or chromosome 19 is >=3 and the position at which LOD is maximized falls within insulin QTL region. We permutation gene expression in each tissue and repeat the process 50 times to estimate FDRs. To filter out genes that are independent of insulin, we adopt Bayesian Network approach similar to the causality test developed by Schadt et al. ([Schadt, Lamb et al. 2005](#_ENREF_5)). We used a deal package in R ([Bøttcher and Dethlefsen 2003](#_ENREF_1)) to construct a three-node network, namely, genetic marker, insulin and gene expression traits with genetic marker being the root of the network. We consider genes whose expression trait is either parent or child of insulin trait for next step analysis.

***1.3 Construct protein network and prioritize genes***

A global mouse protein-protein interactions were collected as described in ([Tu, Argmann et al. 2009](#_ENREF_7)). Briefly the set of mouse PPIs was obtained by integrating several public (BIND, BioGRID, HPRD, MINT, Reactome, DIP, and IntAct) and commercial (Ingenuity, Proteome, MetaBase, and NetPro) molecular interaction databases. Genes after filtering as being either causal or reactive to insulin within the same tissue are overlaid on to the protein network to generate interaction network as shown in Figure 5. To further prioritize genes, we develop an algorithm called TIE to rank genes in the network. The calculation of TIE score is described in the main text. For the permutation test, we conserve the network topology and simply randomize the association between gene expression and trait (insulin levels). We repeat the process 1,000 times to obtain an empirical distribution of the TIE scores. P-values are determined as the frequencies of a randomized TIE score being equal or great than the actual TIE score for each node.

**References**:

Bøttcher, S. G. and C. Dethlefsen (2003). "deal: A package for learning Bayesian networks." Department of Mathematical Sciences, Aalborg University(394c5886-54d6-0e0f-3fae-4be7c2fafe1f).

Broman, K., H. Wu, et al. (2003). "R/qtl: QTL mapping in experimental crosses." Bioinformatics (Oxford, England) **19**(7): 889-979.

Jiang, C. and Z. Zeng (1995). "Multiple trait analysis of genetic mapping for quantitative trait loci." Genetics **140**(fa9e66e9-b664-7f9b-1031-4b13cb5863a0): 1111-1138.

Keller, M. P., Y. Choi, et al. (2008). "A gene expression network model of type 2 diabetes links cell cycle regulation in islets with diabetes susceptibility." Genome Res **18**(5): 706-716.

Schadt, E., J. Lamb, et al. (2005). "An integrative genomics approach to infer causal associations between gene expression and disease." Nature genetics **37**(7): 710-717.

Stoehr, J. P., S. T. Nadler, et al. (2000). "Genetic obesity unmasks nonlinear interactions between murine type 2 diabetes susceptibility loci." Diabetes **49**(11): 1946-1954.

Tu, Z., C. Argmann, et al. (2009). "Integrating siRNA and proteinâ€“protein interaction data to identify an expanded insulin signaling network." Genome Research **19**(6): 1057-1067.

Zhong, H., J. Beaulaurier, et al. (2010). "Liver and Adipose Expression Associated SNPs Are Enriched for Association to Type 2 Diabetes." PLoS Genet **6**(5): e1000932.
